# Associations between hair-derived cannabinoid levels, self-reported use, and cannabis-related problems

**DOI:** 10.1007/s00213-024-06558-0

**Published:** 2024-02-26

**Authors:** Emese Kroon, Janna Cousijn, Francesca Filbey, Christian Berchtold, Tina M. Binz, Lauren Kuhns

**Affiliations:** 1 Department of Psychology, University of Amsterdam, Amsterdam, The Netherlands; 2 Neuroscience of Addiction (NofA) Lab, Center for Substance Use and Addiction Research (CESAR), Department of Psychology, Education & Child Studies, Erasmus University Rotterdam, Rotterdam, The Netherlands; 3 Department of Psychology, School of Behavioral and Brain Sciences, University of Texas at Dallas, Dallas, TX USA; 4 Center for Forensic Hair Analytics, Zurich Institute of Forensic Medicine, University of Zurich, Zurich, Switzerland

**Keywords:** Cannabis, Cannabinoids, Self-report, Hair analysis

## Abstract

**Rationale:**

As cannabis potency and cannabis use are increasing in newly legalized markets, it is increasingly important to measure and examine the effects of cannabinoid exposure.

**Objectives:**

The current study aims to assess how hair-derived cannabinoid concentrations – offering insight into three-month cumulative exposure – are associated with common self-report measures of cannabis use and cannabis use-related problems.

**Methods:**

74 near-daily dependent cannabis users self-reported their quantity of cannabis use, cannabis use-related problems, and estimated cannabis potency. Hair samples were provided to quantify Δ9-THC, CBD, and CBN using LC–MS/MS and THC-consumption was verified by analyzing THC-COOH in hair using GC–MS/MS.

**Results:**

Cannabinoids were detectable in 95.95% of the hair samples from individuals who tested positive on a urine screen for cannabis. Δ9-THC concentrations were positively associated with measures of self-reported potency (relative potency, potency category, and perceived ‘high’), but Δ9-THC, CBD, CBN concentrations and THC/CBD ratio were not associated with self-reported quantity of use. Self-reported potency, but not hair-derived concentrations, were associated with withdrawal and craving. Self-reported quantity of cannabis use, but not cannabinoid concentrations, were associated with cannabis use-related problems.

**Conclusions:**

The use of hair-derived cannabinoid quantification is supported for detecting cannabis use in near-daily users, but the lack of associations between hair-derived cannabinoid concentrations and self-report measures of use does not support the use of hair analyses alone for quantification of cannabinoid exposure. Further research comparing hair-derived cannabinoid concentrations with other biological matrices (e.g. plasma) and self-report is necessary to further evaluate the validity of hair analyses for this purpose.

**Supplementary Information:**

The online version contains supplementary material available at 10.1007/s00213-024-06558-0.

## Introduction

Cannabis is the most widely used drug with more than 209 million past year users (United Nations Office on Drugs and Crime 2021). Given the evidence of increasing use in newly legalized markets (Hall and Lynskey 2020) and parallel increases in cannabis potency (United Nations Office on Drugs and Crime 2022), it is critical to examine the effects of cannabis use on health. Measuring cannabinoid exposure presents a uniquely complicated challenge, given the variation in the cannabinoid content of products and differences in bioavailability depending on route of administration. Hair analysis may provide a relatively accessible non-invasive method to complement self-reports to investigate the effects of cannabinoid exposure on health. However, it is currently unclear how suitable hair analysis is for quantifying cumulative cannabinoid exposure in frequent users. The aim of the current study was to examine the associations between different self-reported measures of cannabis use and hair-derived analysis of cumulative cannabinoid exposure with measures of cannabis-related problems to guide the selection of measures in future cannabis research.

The iCannToolkit was recently proposed by a consensus of international cannabis experts to standardize the measurement of cannabis use (Lorenzetti et al. 2021). The framework consists of three layers of assessment that differ in their accessibility and level of detail. The universal base layer is suitable for quick assessment in population-based surveys and emergency service settings and proposes using three self-report items to assess ever use, last use, and days of cannabis use in the past month. The mid layer is suitable for in-depth research on the effects of cannabis use on health and proposes detailed self-report assessment using the timeline followback methodology (TLFB; Sobell and Sobell 1992) to assess the quantity of use per day over a specific period of time (i.e. past week, past month). However, inherent difficulties in accurately measuring cannabis and cannabinoid exposure emerge in this layer. There is substantial variation both within and across individuals in the types of cannabis products used, the method of administration, and the potency of products, which limits the ability to understand the effects associated with the main compounds in cannabis, particularly psychoactive Δ9-THC and non-psychoactive CBD. Experimental evidence suggests a dose–response relationship between THC exposure and related harms (Kroon et al. 2020; Hines et al. 2020), but a detailed investigation of the effects of cannabis exposure in observational research requires the development of more accurate quantification methods. Because of this, the top-layer of the iCannToolkit includes biological measures to quantify cannabinoids or their metabolites in urine, saliva, plasma, or in the cannabis product itself. Several studies found strong correlations between TLFB-reported recent cannabis use and THC and metabolite concentration in urine and plasma (Hjorthøj et al. 2012; Barguil et al. 2022). However, these methods are challenging to use for many researchers and clinicians due to the invasiveness and lack of accessibility (e.g. storage requirements). For example, cannabinoid metabolites lack stability in both urine and plasma samples when stored for even short periods at room temperature, resulting in metabolite degradation and inaccurate measurement (Dugan et al. 1994; Fraga et al. 1998; Skopp and Pötsch 2002). Furthermore, urine and plasma analysis only detect cannabinoid concentrations within a narrow window of time, typically no more than 7 days. Cumulative exposure to cannabinoids over longer periods of time may be more informative regarding the effects of cannabis use on well-being, which develop over longer periods of time. While testing cannabis products would be valuable, it is complicated by differences in legal status across jurisdictions and product variability.

Analysis of cannabinoid metabolites in hair samples may be a viable alternative to measure cumulative exposure over longer periods of time. Hair grows, on average, 1 cm per month and therefore the analysis of 1 cm of hair can provide insights into drug consumption during the past month. Additionally, hair sampling is non-invasive, and hair can be stored at room temperature (Musshoff and Madea 2006). This can be beneficial for investigating whether greater cumulative cannabinoid exposure, including THC and other compounds such as cannabinol (CBN), in chronic heavy users translates to increased harm and whether CBD may have protective effects. The state-of-the-art methods to quantify cannabinoid concentrations in hair have developed substantially over time and the used preparation and analysis methods influence the validity of the quantification (Shah et al. 2019). Liquid chromatography-mass spectrometry (LC–MS) is a gold standard method for detection of drugs of abuse, including THC (Shah et al. 2019). In a study of cannabis using psychiatric patients, LC–MS derived THC concentration and THC/CBD ratio were identified as potential markers for acute and chronic psychosis (Barguil et al. 2022).

To our knowledge, no studies have yet investigated the associations between TLFB reported recent cannabis use (the mid-layer of the iCannToolkit), cannabis use-related problems, self-reported potency of typically used products, and hair-derived measures using liquid chromatography- tandem mass spectrometry (LC–MS/MS). Therefore, we aimed to assess how self-report measures of cannabis use, use-related problems, and potency are associated with each other and with hair-derived THC, CBD, CBN, and THC/CBD concentrations from the previous three months.

## Methods & materials

### Participants

Seventy-four cannabis users completed the included assessments as part of a larger fMRI project (Kroon et al. 2023). The study was approved by the ethics committee of the Department of Psychology of the University of Amsterdam (2018-DP-9616). All participants were between 18–31 years old, used cannabis 6–7 days per week on average for at least the previous year, had a mild-to-severe cannabis use disorder (MINI CUD score > 1; Sheehan et al. 1997), did not seek treatment for their CUD, had no current psychological diagnoses other than anxiety, depression or ADHD/ADD, and did not use psychotropic medication.

### Measures

#### Questionnaires

Participants reported their age and sex. Cannabis use-related problems were assessed using the Marijuana Problem Scale (MPS; Hodgins and Stea 2018), Cannabis Use Disorder Identification Test (CUDIT-R; Adamson et al. 2010), CUD semi-structured interview from the Mini International Neuropsychiatric Interview (MINI; Sheehan et al. 1997), Marijuana Withdrawal Checklist (MWC; Budney et al. 1999), Marijuana Craving Questionnaire (MCQ; Heishman et al. 2009), and a craving Visual Analogue Scale (VAS; Mottola 1993). Cannabis use was assessed using a one-month Timeline Follow Back questionnaire (Sobell and Sobell 1992; Robinson et al. 2014) and with self-reported grams per week (days per week x grams per use day). Self-report measures of cannabis potency included price per gram, relative potency (scale 0–100), potency (category – very mild/mild/average/strong/very strong), perceived ‘high’ (scale 1–5), and THC percentage (categorical; see full questions in figure S1). Participants also reported their preferred type of cannabis (flower/concentrate) and whether they regularly added tobacco to their cannabis (yes/no) when smoking it. Measures of other drug use included daily cigarette use (yes/no), the Fagerström Test for Nicotine Dependence (FTND; Heatherton et al. 1991), the Alcohol Use Disorder Identification Test (AUDIT; Saunders et al. 1993), and self-reported lifetime use of any drugs besides cannabis, alcohol, and tobacco.

#### Urine and hair samples

The presence (yes/no) of THC metabolites was assessed in urine (threshold 50 ng/mL THC-COOH). Hair was taken from the nape of the neck and sent to the Centre for Forensic Hair Analysis at the University of Zurich. A liquid chromatography atmospheric pressure chemical ionization-tandem mass spectrometry (LC-APCI-MS/MS) method was used for quantification of Δ9-THC, CBN and CBD in the 3 cm long hair samples (pg/mg; Scholz et al. 2022). Δ9-THC and CBD concentrations were used to calculate THC/CBD concentrations. A gas chromatography electron impact-ionization-tandem mass spectrometry method, using BSTFA derivatization, was used to analyze THC-COOH in hair (see Supplementary Materials). After LC–MS/MS analysis for THC, sample extracts were re-analyzed for THC-COOH to confirm THC consumption (Franz et al. 2018).

### Data analysis

Non-parametric Kendall’s tau correlations, fit for non-normal and ordinal data, were performed to assess the associations between 1) measures of cannabis use-related problems, 2) self-reported cannabis use outcomes calculated from the TLFB (gram/day and days of use for 1 month, 14 days, and 7 days), and 3) hair-derived cannabinoid concentrations cumulated over the past three months. Due to the exploratory nature of this study, we did not correct for multiple comparisons but provided Bayes factors to be able to evaluate the strength of the evidence (Jeffreys 1961) for the significant correlations (H_0_: no correlation; Bayes Factor (BF_10_) > 100: extremely strong evidence for H_a_, BF_10_ 30–100: very strong evidence for H_a_, BF_10_ 10–30: strong evidence for H_a_, BF_10_ 3–10: moderate evidence for H_a_). Correlations were interpreted as significant if the Kendall’s tau correlation was significant (*p* < 0.05) and there was at least moderate evidence for the correlation (BF_10_ > 3.00). Individuals who tested positive for THC in urine screening but negative for cannabinoids in hair analyses were excluded from the analyses (*N* = 2). We conducted sensitivity analyses excluding outliers > 2 SD above the mean cannabinoid concentrations (THC > 2SD = 4, CBD > 2SD = 5, CBN < 2SD = 7). Additionally, we excluded values based on minimum thresholds used in legal proceedings in the detection of cannabis use (THC < 50 = 33, CBD < 50 = 46, CBD < 50 = 35). We only reported effects that remained significant in these sensitivity analyses. Analyses were conducted using JASP version 0.16.4.0 (JASP Team 2022).

## Results

### Sample characteristics

All 74 participants (66.22% male) tested positive for THC on the urine screening, with 72 participants (95.95%) also testing positive for THC in hair (Table 1). For 40 from the 72 THC positive participants the THC-consumption was confirmed by detecting THC-COOH (see Table S2 for additional information). In one sample with very low THC concentration (< 20 pg/mg) THC-COOH was not detected31 THC-positive participants could not be re-analyzed for THC-COOH due to insufficient sample volume.. Additionally, 4 samples from the control group (75% male, mean age = 21.5, 75% ever used cannabis, no use within last month and no more than 3 use occasions in last year, mean lifetime use occasions in ever users = 3.33) were analyzed and showed no THC-COOH, as expected. Participants used a median of 6 g in an average week, reporting between 13 and 31 days of cannabis use (median = 30) and using a little less than 1 g (median = 0.87) per day during the last month. CUDIT-R scores (median = 16) were indicative of problematic use (score > 12; Adamson et al. 2010). The use of flower products (64.87%) was more common than the use of concentrates (35.13%), with no individuals reporting a preference for other products. Together, the self-report measures of potency were indicative of average-strong perceived potency and experienced ‘high’. Half of the participants reported daily cigarette use, with variable levels of nicotine dependence (FTND range: 1–7, median = 5), and 93.06% reported regularly adding tobacco to their cannabis. AUDIT scores (median = 5) were below at-risk alcohol use (score > 8), but 2.7% (*N* = 2) of participants reported potential hazardous use (score > 12; Saunders et al. 1993).

**Table 1:** Sample characteristics

Scale and ordinal outcomes	Description	Median (MAD)	Range	N
General				
Age	years	21 (2)	18–31	74
Cannabis use				
Average cannabis use	Gram/week	6 (3.2)	.28–21.00	71
Cannabis use days (TLFB)	Last month	30 (1)	13–31	70
	Last 14 days	13 (1)	6–14	69
	Last 7 days	6 (1)	2–7	70
Cannabis gram/day (TLFB)	Last month	.87 (.32)	.07–3.00	70
	Last 14 days	89 (.38)	.03–3.00	70
	Last 7 days	.85 (.44)	.05–2.86	70
Cannabis use age of onset	years	15 (1)	12–19	72
Cannabis use-related problems				
Cannabis Use Disorder symptoms	MINI CUD score	5 (1)	2–10	74
Cannabis use problems	MPS score	6.5 (3.5)	0–32	74
Cannabis use and related problems	CUDIT-R score	16 (5.0)	6–32	74
Withdrawal	MWQ score	8 (3)	1–25	74
Craving	MCQ score	40.5 (9.5)	16–76	74
	VAS score	5.5 (1.5)	0–9.6	74
Other drug use				
Alcohol use and related problems	AUDIT score	5 (2)	1–14	73
Nicotine dependence	FTND score	5 (1)	1–7	37
Cigarette use	Cigarettes/day	7 (3)	2–21	37
Other drug use	Lifetime	13.5 (13.5)	0–352	74
Self-reported potency estimates				
Self-reported relative potency	Scale 0–100	65 (15)	0–100	74
Self-reported ‘high’	Scale 1–5	4 (1)	1–5	74
Self-reported price per gram	Euro	9.5 (1.5)	3–15	73
Cannabinoids in hair				
THC	pg/mg	62.00 (45.00)	6–3200	71
CBD	pg/mg	38.00 (22.00)	10–1900	71
CBN	pg/mg	56.00 (31.00)	11–1800	71
THC/CBD	pg/mg	1.33 (1.25)	.03–36.36	71
Nominal outcomes	Description	Percentage		N
Gender	F/M	33.78/66.22		74
Urine screening THC	Positive/negative	100.00/0.00		74
Daily cigarette use	yes/no	50.00/50.00		74
Preferred cannabis type	concentrate/flower	35.13/64.87		74
Tobacco added to cannabis	yes/no	93.06/6.94		72
Self-reported potency	very light/light/average/strong/very strong	0.00/1.35/50.00/36.49/12.16		74
Self-reported THC percentage	< 5/5–10/10–15/15–20/20–25/25–30/ > 30	0.00/5.41/20.27/40.54/28.38/4.05/1.35		74

*TLFB* Timeline follow back; *THC* Delta-9-tetrahydrocannabinol; *CBD* Cannabidiol; *CBN* Cannabinol; *MINI CUD* Mini international neuropsychiatric interview, cannabis use disorder; *MPS* Marijuana problem scale; *CUDIT-R* Cannabis use disorder identification test; *MWQ* Marijuana withdrawal questionnaire; *MCQ* Marijuana craving questionnaire; *VAS* Visual analogue scale; *AUDIT* Alcohol use disorder identification test; *FTND* Fagerström test for nicotine dependence; *pg/mg* picogram per milligram; *ms* milliseconds

### Measures of cannabis use, cannabis use-related problems and potency

There was decisive evidence for a positive correlation of THC and CBD hair concentrations with hair CBN, but no evidence for a correlation between hair THC and hair CBD concentrations or between THC/CBD ratio and CBN (Table 2). Furthermore, there was moderate to strong evidence for a positive correlation between THC concentrations and self-reported relative potency, perceived ‘high’, and potency (category) with strong evidence for a similar correlation between CBN concentrations and potency (category). Cannabinoid concentrations were not associated with other measures of cannabis use and related problems.

**Table 2:**
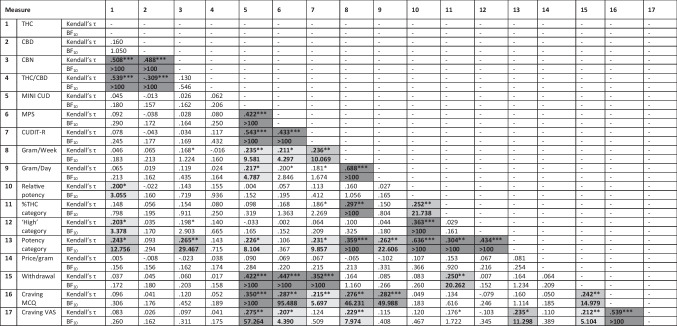
Correlations between measures of cannabinoids, cannabis use, cannabis use-related problems and self-reported measures of potency

*H*_*0*_ no correlation; BF_10_ > 100: extremely strong evidence for H_a_, BF_10_ 30–100: very strong evidence for H_a_, BF_10_ 10–30: strong evidence for H_a_, BF_10_ 3–10: moderate evidence for H_a_, BF_10_ 1–3: anecdotal evidence for H_a_, BF_10_ .30–1.00: anecdotal evidence for H_0_, BF_10_ .10-.30 (moderate evidence for H_0_). BF_10_ > 3 are colored in shades of grey with darker colors representing stronger evidence for H_a_; Significance levels: * *p* < .05, ** *p* < .01, ***, *p* < .001; Correlations considered significant based on *p* < .05 and BF_10_ > 3 are presented in bold. THC: delta-9-tetrahydrocannabinol; *CBD* Cannabidiol; *CBN* Cannabinol; *MINI CUD* mini international neuropsychiatric interview, cannabis use disorder; *MPS* Marijuana problem scale; *CUDIT-R*: Cannabis use disorder identification test; *MCQ* Marijuana craving questionnaire; *VAS* Visual analogue scale

Self-reported relative potency and THC percentage (category) were positively correlated with cannabis use in gram/week (decisive evidence), with only relative potency showing a similar correlation with gram/day in the last month (strong evidence). There was moderate evidence for a positive correlation between potency (category) and CUDIT-R score, whereas no correlations between other measures of cannabis use-related problems and self-reported potency were observed. There were several positive correlations among the different self-report measures of potency (Table 2), but no correlations with price per gram were observed. Furthermore, there was a strong positive correlation between self-reported THC percentage (category) and withdrawal, as well as craving (VAS) and self-reported potency (category).

There was decisive evidence for a positive correlation between CUD, MPS and CUDIT-R scores, and moderate to strong evidence for a positive correlation of those measures with cannabis use in gram/week. The measure of gram/day based on last month TLFB assessment only showed anecdotal to moderate positive correlations with CUD, MPS and CUDIT scores. There was decisive evidence for a positive correlation of CUD, MPS, and CUDIT-R scores with withdrawal, whereas evidence for positive correlations with craving (MCQ and VAS) was mixed depending on the measure of cannabis use-related problems (Table 2). However, while there was no evidence for a correlation between withdrawal and measures of cannabis use (gram/week or gram/day), there was decisive evidence for a positive correlation of those measures with craving (MCQ). Furthermore, there was decisive evidence for a positive correlation between both measures of craving, and moderate to strong evidence for positive correlations between those measures and withdrawal.

Looking at the correlations between different outcomes calculated from the last month TLFB and self-reported gram/week (Table 3), there was very strong to decisive evidence for positive correlations between all measures, regardless of timeline (1 month, 14 days, 7 days) and unit (number of days, gram/day).

**Table 3:**
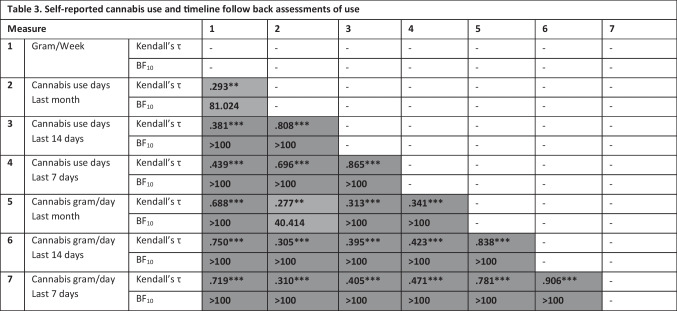
Self-reported cannabis use and timeline follow back assessments of use

*H*_*0*_ no correlation; BF_10_ > 100: extremely strong evidence for H_a_, BF_10_ 30–100: very strong evidence for H_a_, BF_10_ 10–30: strong evidence for H_a_, BF_10_ 3–10: moderate evidence for H_a_, BF_10_ 1–3: anecdotal evidence for H_a_, BF_10_ .30–1.00: anecdotal evidence for H_0_, BF_10_ .10-.30 (moderate evidence for H_0_). BF_10_ > 3 are colored in shades of grey with darker colors representing stronger evidence for H_a_; Significance levels: * *p* < .05, ** *p* < .01, ***, *p* < .001. Correlations considered significant based on *p* < .05 and BF_10_ > 3 are presented in bold

## Discussion

The aim of this study was to examine how self-report measures of cannabis use and potency and hair-derived quantifications of cumulative cannabinoid exposure in individuals with CUD relate to each other and self-reported measures of use-related problems to guide recommendations for cannabis and cannabinoid measures in future research. While self-reported quantity of use was not associated with cannabinoid concentrations, some measures of self-reported perceived potency were positively associated with hair-derived THC and CBN concentrations. The lack of associations between cannabinoid concentrations and TLFB self-reported use and cannabis-related problems does not provide support for the use of hair analysis for quantification of cumulative cannabis exposure in near-daily users.

Hair-derived cannabinoids were detected in 95.95% of cannabis users who met the diagnostic criteria for CUD and tested positive for cannabis in a urine sample, indicating the utility of hair analysis for yes/no detection of cannabis use in heavy users, aligning with Steinhoff and colleague’s findings indicating high agreement between self-report weekly or daily use with detection in hair (Steinhoff et al. 2023). Cannabinoid concentrations were not related to measures of cannabis-related problems or grams per day as measured by the TLFB or self-reported grams per week. While variability in product potency could weaken correlations between self-reported cannabis use and cannabinoid exposure, the previously observed strong correlations between blood plasma-derived cannabinoids and self-reports (Hjorthøj et al. 2012; Barguil et al. 2022) suggest that limitations related to hair analysis should also be considered. Factors such as environmental contamination (Moosmann et al. 2015; i.e. smoke, transfer from other via sebum/sweat; Berthet et al. 2016) likely introduce noise into the data which may obscure associations and different cannabinoid extraction methods might affect comparability across studies. Quantification of THC metabolites instead of cannabinoids themselves would circumvent the issue of environmental contamination but is practically and technically challenging (Moosmann et al. 2015). Furthermore, individual factors can influence the bioavailability and metabolism of cannabinoids, including but not limited to sex, frequency of use, and route of administration, further obscuring potential associations. However, we did observe moderate to strong evidence of weak associations of both THC and CBN concentrations with self-reported perceived potency of cannabis products. While this suggests there is an observable signal in the hair of near-daily cannabis users, it does not justify its use for cannabinoid quantification given the described drawbacks.

Importantly, TLFB-derived grams per day based on either a 7-, 14-, or 31-day period were highly associated and showed similar associations with other measures. While additional studies are needed to draw strong conclusions about the validity of different time frames, the results suggest that even the 7-day TLFB is a valuable measure of cannabis use that can be administered quickly in line with the mid-layer of the iCannToolkit. Grams per week, calculated based on the two-item self-report of typical days of use per week and typical grams per day, was more strongly and consistently related to cannabis use-related problems than the TLFB-derived grams per day measures. Given the short length, the validity and reliability of this measure should be further investigated as it may be flexibly implemented in large scale epidemiological studies of the effects of cannabis use on physical and mental health.

A few limitations are important to discuss. First, these findings are specific to a sample of Dutch individuals who meet the diagnostic criteria for CUD. Suitability of hair-derived cannabinoid quantification may differ depending on severity of use, with detection potentially more difficult in more occasional users (e.g. Taylor et al. 2017). Additionally, the included sample consisted only of individuals who use cannabis flower or concentrates. While the specificity of the sample removed noise that would be introduced via different cannabis products and methods of administration, it also limits the generalizability of the findings. Furthermore, age differences in hair-derived cannabinoid concentrations should be considered in future studies—requiring the inclusion of a larger age range – and sex differences – potentially dependent on differences in hair treatment – should be assessed in studies with larger sample sizes (Vaiano et al. 2023). Finally, the absence of other biospecimens to compare to the hair-derived cannabinoid concentrations limits the strength of the conclusions we can draw about both the suitability of the method and the validity of the associations between self-report use measures, and potency. Future studies including the iCannToolkit proposed plasma, urine, saliva, and cannabis products themselves in addition to hair are crucial for a clear determination of the value of hair analysis and the reliability of biospecimen analyses generally.

In conclusion, the use of hair-derived cannabinoid quantification is supported for detecting cannabis use in heavy, near-daily users, with a 95.95% overlap with cannabis use detection in urine. However, the lack of correlations between cannabinoid concentrations and self-reported use and problems suggests it is not currently a suitable method for quantifying the level of cumulative cannabis exposure in the previous three months.

### Supplementary Information

Below is the link to the electronic supplementary material.

Supplementary file1 (PDF 294 KB)

## Data Availability

The data, code and materials of this study are available from the corresponding author upon reasonable request.

## Declarations

### Preprint

A preprint version of this article is available at https://psyarxiv.com/s6rn2

### Preregistration

The study design and the analysis plans were not preregistered.
