# Supplementary material for: Associations between hair-derived cannabinoid levels, self-reported use, and cannabis-related problems
Source: Psychopharmacology (Berl). 2024 Feb 26;241(6):1237–44. doi: 10.1007/s00213-024-06558-0 (PMC11106191; doi:10.1007/s00213-024-06558-0)
Supplement: Supplementary file 1 — Supplementary file1 (PDF 294 KB) [file 213_2024_6558_MOESM1_ESM.pdf]

Supplementary Materials

Associations between hair-derived cannabinoid levels, self-reported use, and cannabis-related problems

Emese Kroon[1,2], Janna Cousijn[1,2], Francesca Filbey[3], Christian Berchtold[4], Tina M. Binz[4], Lauren Kuhns[1]

[1] Department of Psychology, University of Amsterdam, the Netherlands
[2] Neuroscience of Addiction (NofA) Lab, Center for Substance Use and Addiction Research (CESAR), Department of Psychology, Education & Child Studies, Erasmus University Rotterdam, The Netherlands
[3] Department of Psychology, School of Behavioral and Brain Sciences, University of Texas at Dallas, Dallas, TX, USA
[4] Center for Forensic Hair Analytics, Zurich Institute of Forensic Medicine, University of Zurich, Switzerland

Correspondence: Emese Kroon, emesekroon@gmail.com, P.O. box 15916, 1001 NK Amsterdam, The Netherlands

Declarations of interest: none

Funding: This research was supported by grant 1R01 DA042490-01A1 awarded to Janna Cousijn and Francesca Filbey from the National Institute on Drug Abuse/National Institute of Health.

Author contributions: All authors reviewed and approved the final version. *Conceptualization:* EK, JC & LK; *Methodology:* EK, JC, CB, TMB, LK; Investigation: EK & LK; *Formal Analysis:* EK, CB & TMB; *Data curation:* EK & TMB; *Writing – Original draft:* EK & LK; *Writing – Review & Editing:* JC, FF, CB & TMB; *Visualization:* EK; *Supervision:* JC; *Funding Acquisition:* JC & FF.

Preregistration. The study design and the analysis plans were not preregistered.

Data Accessibility: The data, code and materials of this study are available from the corresponding author upon reasonable request.

Price per gram

How much does the cannabis you typically consume cost? Please state per gram.

……….. per gram

Relative potency

When comparing it to other types of cannabis you have used, how potent is the cannabis you typically use?

0--100

Perceived ‘high’

How strong is the ‘high’ you get from the cannabis you typically use?

(not strong at all) 1 -- 5 (very strong)

Potency category

Please categorize the potency of the cannabis that you typically use.

- Very mild
- Mild
- Average
- Strong
- Very Strong

THC percentage category

How much THC does the cannabis you typically use contain?

- 0-5%
- 5-10%
- 10-15%
- 15-20%
- 20-25%
- 25-30%
- More than 30%

Figure S1. Overview of self-report measures of potency

Analysis of THC-COOH in hair samples with GC-MS/MS

Chemicals:

Methanol LC-MS grade, ethyl acetate LC-MS grade, N,O-Bis(trimethylsilyl)trifluoroacetamide with 1% trimethylchlorsylane (BSTFA, 1% TMCS) and 11-nor-9-carboxy-Δ9-THC were obtained from Merck (Darmstadt, Germany).

Instrumentation:

An Agilent (Santa Clara, CA, USA) 7890B GC-System equipped with a split/splitless injector, a CTC Analytics (Zwingen, CH) PAL LSI 85 autosampler, and an Agilent 7000C MS Triple Quad with EI ionization were used (using Nitrogen as collision gas). Separation was performed on an Agilent HP-5ms Ultra Inert Column (30m, 0.25mm 0.25 µm) using helium as carrier gas.

Method Details:

GC-MS/MS method parameter:

Derivatisation

The validation for THC-COOH with GC-MS/MS was performed for the standard method, when 750 µL extract was available for hair extraction. LOD and LOQ for this method were 0.2 pg/mg and 1 pg/mg respectively (see table S1).

However, for the retrospective measurement of sample extracts for THC-COOH only 100 µL hair extract were available, which were diluted with buffer 1:1 for the initial LC-MS/MS analysis. THC-COOH analysis by GC-MS/MS was performed with this remaining extract. To extrapolate concentrations and do semi-quantitation a calibration was performed in hair sample extracts (100 µL), which were prepared the same way as the samples (see Figure S2). For this method LOD and LOQ were also determined and found to be 5 pg/mg and 50 pg/mg (Table S1).

- **750 µL** methanolic sample extract (from previous extraction) was dried under nitrogen (60 min. at 35°C)
- Sample extracts after LC-MS/MS analysis (**100 µL** extract and **100 µL** buffer) were dried under nitrogen (60 min. at 35°C)
- 50 µL BSTFA was added to the dried extracts and derivatization was performed for 30 min. at 80°C
- The samples were then transferred to a silanized insert (300 µL) and injected into the GC-MS/MS

GC-MS/MS-method parameters

Rate °C/min	Temperatur	Hold Time
0	150	1
10	300	4

Multiple reaction monitoring mode (MRM):

Time Segment	Cannabinoid	Precursor Ion	Product Ion	Collision Energy [V]
13.43-13.65	Δ9-THC-COOH	488.1	419.8	25
		488.1	366.0	25

Limit of detection (LOD) and limit of quantification (LOQ)

Table S1. LOD and LOQ determined for the different sample extracts		
**Sample extraction volume**	LOD<br>pg/mg	LOQ<br>pg/mg
THC-COOH<br>(750 µL hair sample extract)	0.2	1
THC-COOH<br>(LC-MS/MS sample extract 100 µL+ 100 µL buffer)	5	50

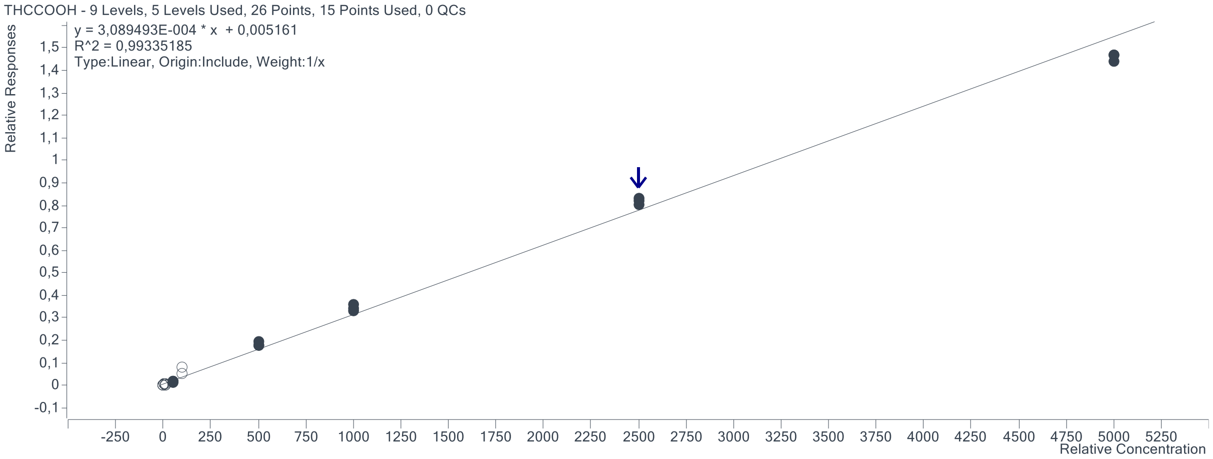

Figure S2. Linearity of THC-COOH concentration from LC-MS/MS sample extracts.The calibration was done in spiked LC-MS/MS samples extracts (100 µL spiked extract diluted with 100 µL buffer, dried and derivatized).

Calibration Range:

	**Cal 1 pg/mg**	**Cal 2 pg/mg**	**Cal 3 pg/mg**	**Cal 4 pg/mg**	**Cal 5 pg/mg**
THC-COOH	50	500	1000	2500	5000

Recovery

The recoveries were determined by measuring spiked blank hair samples at two concentrations and compared to the values with a pure standard.

- At 1000 pg/mg, 83.3%
- At 100 pg/mg, 112.1%

**Table S2.** THC-COOH concentrations analysed in cannabis and control group		
**Randomized Sample Identifier**	**THC-COOH in pg/mg**	**Group**
1	< LOD	Control
2	< LOD	Control
3	< LOD	Control
4	< LOD	Control
5	< LOD	Cannabis
6	8	Cannabis
7	8	Cannabis
8	9	Cannabis
9	10	Cannabis
10	10	Cannabis
11	13	Cannabis
12	13	Cannabis
13	13	Cannabis
14	16	Cannabis
15	16	Cannabis
16	21	Cannabis
17	30	Cannabis
18	33	Cannabis
19	35	Cannabis
20	40	Cannabis
21	41	Cannabis
22	47	Cannabis
23	48	Cannabis
24	65	Cannabis
25	66	Cannabis
26	72	Cannabis
27	79	Cannabis
28	80	Cannabis
29	83	Cannabis
30	107	Cannabis
31	125	Cannabis
32	149	Cannabis
33	173	Cannabis
34	207	Cannabis
35	213	Cannabis
36	302	Cannabis
37	370	Cannabis
38	375	Cannabis
39	429	Cannabis
40	473	Cannabis
41	495	Cannabis
42	679	Cannabis
43	854	Cannabis
44	1507	Cannabis
45	1525	Cannabis
46	1654	Cannabis
47	2724	Cannabis
